# COVID-19 Impact on Smokers Participating in Smoking Cessation Trials: The Experience of Nondaily Smokers Participating in a Smartphone App Study

**DOI:** 10.1089/tmr.2021.0008

**Published:** 2021-06-14

**Authors:** Susanne S. Hoeppner, Hannah A. Carlon, Christopher W. Kahler, Elyse R. Park, Audrey Darville, Damaris J. Rohsenow, Bettina B. Hoeppner

**Affiliations:** ^1^ OCD and Related Disorders Program, Department of Psychiatry, Massachusetts General Hospital, Boston, Massachusetts, USA.; ^2^ Department of Psychiatry, Recovery Research Institute, Massachusetts General Hospital, Boston, Massachusetts, USA.; ^3^ Center for Alcohol and Addiction Studies, Brown University School of Public Health, Providence, Rhode Island, USA.; ^4^ Department of Psychiatry, MGH Health Policy Research Center, Massachusetts General Hospital, Boston, Massachusetts, USA.; ^5^ College of Nursing, University of Kentucky, Lexington, Kentucky, USA.

**Keywords:** COVID-19, nondaily, positive psychology, smoking cessation, stress

## Abstract

**Objectives:** To provide initial insight into how the COVID-19 pandemic could affect smoking behaviors and cessation efforts that were underway at its onset.

**Methods:** An additional survey was added to follow-up assessments in an ongoing smoking cessation study for nondaily smokers: a measure of impact of COVID-19 and a subset of previously administered scales measuring smoking, emotional well-being, and alcohol use. Pre–post tests were conducted (84 ± 28 days apart).

**Results:** Participants (81/100 of enrolled; 67% female, 75% white, 10% Hispanic, 37 ± 11 years old) reported experiencing changes regarding work (35% income reduction/loss; 35% remote work) and living situation (15% consolidated residences). Participants reported their motivation to quit smoking “slightly” increased after COVID-19 (*p* < 0.001), more so in those having achieved 30-day abstinence (*p* = 0.0045). Worry, fear, and a desire to support the greater good increased (*p*s < 0.05). Increases in motivation to quit correlated positively with prosocial and wellness changes. Data from pre- to post-COVID-19 onset showed decreases in emotional well-being (increased stress, negative affect, decreased coping, positive affect, all *p*s < 0.01), but not changes in smoking abstinence (*p* = 0.65), readiness to quit (*p* = 0.16), smoking frequency (*p* = 0.96), or cigarettes per day (*p* = 0.96). Heavy drinking decreased (*p* < 0.01). Trying e-cigarettes increased (*p* = 0.04).

**Conclusions:** Nondaily smokers participating in a smoking cessation study during the COVID-19 pandemic reported worsened emotional well-being without effects on smoking outcomes and said their motivation to quit was slightly increased. Correlations of motivation to quit with prosocial and wellness changes suggest that targeting these constructs may be particularly helpful during a pandemic.

## Introduction

The COVID-19 pandemic and resulting social distancing could affect smoking behaviors and cessation efforts that were underway at its onset. Understanding this impact may be useful to clinical practice efforts and can provide context for ongoing smoking cessation trials.

A critical influence to consider is mental health changes. The COVID-19 pandemic and social distancing efforts are expected to have substantial impacts on the general population's mental health. Early reports from China,^1,2^ Australia,^3^ and the United States^4^ found that many reported increased psychological distress during the early months of the pandemic, including increases in loneliness, depression, anxiety, and stress symptoms. Because strong evidence links the experience of stress to smoking behaviors,^5–7^ concerns about pandemic-related increases in negative emotions leading to increased smoking are highlighted on the Centers for Disease Control and Prevention (CDC) COVID-19-specific website.^8^ Indeed, a survey of nicotine users spanning five countries affected by COVID-19 (Italy, India, South Africa, the United Kingdom, and the United States) found that nicotine products were the primary stress and anxiety coping tool for users during this time,^9^ and many health care workers fear that the negative mental health consequences resulting from the COVID-19 pandemic will result in the relapse of those who have quit smoking in the past.^10^ Moreover, reports of smoking having a protective effect against contracting COVID-19^11^ may further undermine smoking cessation efforts. Thus, there is strong reason to expect smoking cessation efforts to be adversely affected by the COVID-19 pandemic.

On the counterbalance are several factors through which the COVID-19 pandemic may have favorable impacts on smoking cessation efforts. First, new information suggesting a detrimental impact of smoking on COVID-19 disease progression can motivate smoking cessation. Smoking is among the most prevalent underlying diseases among COVID-19 patients who need hospitalization,^12^ and smoking seems associated with negative progression and adverse outcomes of COVID-19.^13^ In a survey of adult smokers in the United States early in the pandemic (April 10, 2020), participants reported a moderately strong belief that their use of tobacco cigarettes or electronic cigarettes increased their risk of harm from COVID-19, and this belief was positively correlated with motivation to quit.^14^ Second, for many, COVID-19 is a time of financial distress, and financial limitations may inadvertently promote smoking cessation or reduced consumption.^15^ Third, stay-at-home orders may decrease access to tobacco retailers.

Effects of social distancing are more complex. Smokers are especially likely to smoke when socializing^16^ or exposed to the smoking behavior of others.^17,18^ They are less likely to smoke in the presence of others who are not smoking.^19^ Indeed, increased time in smoke-free home environments can lead to increased cessation and reduced smoking.^20^ Thus, during the pandemic, the smoking status of household members and whether residences are smoke-free homes are likely to be influential.

To provide more insight, we conducted a survey with nondaily smokers participating in a smoking cessation study during the first outbreak of the COVID-19 pandemic in the United States. Nondaily smokers have a 72% higher mortality risk compared with never smokers,^21^ with nondaily smoking more common among ethnic minority groups^22^ and persons with mental health and substance use challenges,^23^ both populations disproportionally affected by the COVID-19 pandemic.^24,25^ In this article, we (1) examine nondaily smokers' own estimation of the impact of the COVID-19 pandemic on their emotional state and motivation to quit smoking, and (2) provide pre–post comparisons of smoking status, emotional well-being, and alcohol use before and after the outbreak.

## Materials and Methods

### Participants

Participants were nondaily smokers participating in a single-arm prospective feasibility study of a smoking cessation smartphone app using a positive psychology approach (NCT03951766), who agreed to participate in an optional survey assessment shortly after the first peak of the COVID-19 pandemic in the United States. For the original study, participants needed to be 18 years of age, smoke at least weekly, but no more than 25 out of the past 30 days, own a smartphone, and be ready to make a quit attempt in the study. This study was conducted completely remotely, and participants were recruited nationwide (United States) through social media advertising and posting in clinical trial networks. To enroll, participants had to pass a screening procedure (i.e., phone screen, online baseline survey with check items, provision of contact information for collaterals, and setting of a quit date) and complete an onboarding call 8 days in advance of the chosen quit day. Out of the *n* = 100 participants who enrolled in the original study, *n* = 81 (81%) participated in the COVID-19-specific survey (April 8–26, 2020). Of those who did not participate in the COVID-19-specific survey (*n* = 19; 19%), 6 were reached but actively declined, 10 did not respond to our survey outreach (5 of them had completed the original study at this point), and 3 started the survey but did not proceed beyond providing informed consent. Pre-COVID data were collected between September 3, 2019 and February 27, 2020.

### Procedure

Participants were invited to complete an optional additional survey to capture their experiences since the COVID-19 outbreak. The survey included a COVID-19-specific measure and a subset of previously administered measures of smoking, emotional well-being, and substance use. Participants received $15 for complete or $10 for incomplete surveys. All study procedures were approved by the Partners HealthCare Institutional Review Board. Data were collected and managed using REDCap electronic data capture tools.^26^

### Measures

#### Demographics

At baseline, participants reported on their age, gender, race, ethnicity, education, employment status, and region of residence within the United States.

#### Smoking related

The term “smoking” in this article refers to the use of combustible cigarettes. At all assessments, if participants reported smoking abstinence, they were asked if they had been abstinent for the past 30 days. If they reported any smoking, they were asked if they were seriously thinking of quitting smoking within the next 30 days, 6 months, or not at all, coded into a binary variable of “ready to quit” (i.e., in preparation, action, or maintenance) versus “not” (i.e., precontemplation and contemplation).^27^ They were asked to indicate the number of days they had smoked in the past 30 days and the number of cigarettes they smoked on average per smoking day. Participants indicated if they had ever used e-cigarettes (yes/no), and if yes, if they were currently using them. At baseline, they were asked if they had ever smoked daily and if they had ever tried to quit smoking.

#### COVID-19 impact

Impact of the COVID-19 pandemic on life circumstances and changes in emotions and behaviors were assessed using a COVID-19 Impact Scale we designed (Supplementary Appendix SA1). Participants identified the date when the COVID-19 pandemic “made a noticeable impact on your life,” and then rated 24 items to indicate “since then, have you done/experienced the following things more, less, or about the same as compared to the time during the month before that date?,” using a 7-point Likert scale ranging from −3 (*much less*) to 3 (*much more*). The item of primary interest was “been motivated to quit smoking/stay quit.” Other items assessed emotional well-being, health behaviors, and overall outlook, where our goal was to capture experiences that early reports suggested would be impacted by the pandemic (e.g., stress and anxiety) as well as experiences that may ameliorate such stressors. Participants were then asked to “indicate which of the possible impacts due to the COVID-19 pandemic affected you,” using four check-all-that-apply items: “experienced any changes in your job situation,” “experienced a change in your living situation,” “been impacted by government restrictions,” and “your contact with COVID-19 includes …,” which listed possibilities for personally experiencing COVID-19 (e.g., tested positive and living with someone who tested positive).

#### Emotional well-being

At all assessments, participants completed the following. The 10-item Perceived Stress Scale (PSS-10)^28^ asks about participants' “feelings and thoughts during the last month.” The two-factor^29^ scoring method derived scores for stress and coping, using mean scores of items to relate to the scale participants used to rate items (i.e., *0 = never, 1 = almost never, 2 = sometimes, 3 = fairly often,* and *4 = very often*). The Positive and Negative Affect Schedule (PANAS)^30^ lists 20 adjectives that participants rate “to what extent have you felt each of the following in the past week.” We used mean scores of negative and positive affect.

#### Alcohol use

At all assessments, participants were asked about their alcohol and electronic cigarette use. Participants were asked if they drank any alcohol in the past 30 days (yes/no) and if yes, if they drank 4+/5+ (female/male) drinks per drinking occasion, and during how many weeks of the past 4 weeks they drank 7+/14+ drinks during a single week. We coded a nonzero response to the latter question as exceeding National Institute on Alcohol Abuse and Alcoholism (NIAAA) drinking guidelines^31^ and any 4+/5+ (female/male) drinks per drinking occasion reports as engaging in heavy drinking.

### Analytic strategy

#### Baseline data

We computed descriptive statistics, checked for normality, and conducted *t*- and chi-square tests between survey completers versus noncompleters.

#### COVID-19 Impact Scale

We computed means with standard deviations for each item of the COVID-19 Impact Scale and the percentage of participants reporting no change. We conducted one-sample *t*-tests to investigate change (i.e., mean score different from 0), paired *t*-tests to determine if motivation to quit smoking was impacted significantly differently than other scale items, and Pearson's correlations of motivation to quit smoking/stay quit with other impacts.

#### Pre–post COVID-19 tests

To test for changes from pre- to post-outbreak of COVID-19, we used data from the most recent pre-COVID-19 survey based on the date on which COVID-19 made an impact on the participant's life (i.e., the participant's named date, or 3-12-2020, the date on which the first state enacted statewide school closures, whichever was earlier). We conducted repeated measures linear mixed effects models for normally distributed outcomes and generalized estimating equation analyses with binary distributions and log links for binary outcomes. We included data from all enrolled participants (*n* = 100) in the analyses, using a Maximum Likelihood Approach to handle missing data for those who had not completed the COVID-19 surveys. This likelihood based mixed modeling approach^32^ produces unbiased estimates of effects under missing completely and missing at random patterns of missingness.^33^

## Results

### COVID-19 survey participation

Participants are described in Table 1. Females and those smoking on more days were significantly more likely to complete the COVID-19 survey. Two participants provided an incomplete survey. For most participants, the pre-COVID-19 survey was either the 3- or 6-month follow-up survey (51% and 47%, respectively; for 2%, it was the treatment end survey). On average, the COVID-19 survey occurred ∼3 months after the most recent assessment, and ∼1 month after the date marking the beginning of the COVID-19 impact on participants' lives.

**Table 1.** Sample Characteristics

	Completed survey	No survey	*p*
	*n* = 81	*n* = 19	
	Mean /% (SD)/*n*	Mean/% (SD)/*n*	
Demographics			
Age	36.8 (11.1)	31.9 (11.9)	0.09
Gender (% female)	66.7 (54)	36.8 (7)	0.02
Race (in %)			0.18
White	75.3 (61)	73.7 (14)	
Black	16.0 (13)	5.3 (1)	
Other or unknown	8.6 (7)	21.1 (4)	
Hispanic (in %)	9.9 (8)	21.1 (4)	0.23
Education (in %)			0.61
High school or less	16.0 (13)	10.5 (2)	
Some college	44.4 (36)	57.9 (11)	
BA/BS or higher	39.5 (32)	31.6 (6)	
Employment (% full time)	43.8 (35)	42.1 (8)	
Region of residence within the United States (in %)			0.10
Northeast	30.9 (25)	57.9 (11)	
Midwest	18.5 (25)	5.3 (1)	
South	33.3 (27)	15.8 (3)	
West	17.3 (14)	21.1 (4)	
Smoking characteristics at baseline			
No. of days smoked in past 30 days	15.2 (4.2)	12.3 (5.6)	0.01
No. of cigarettes smoked per smoking day	4.8 (4.0)	3.9 (2.9)	0.32
Ever smoked daily? (% yes)	67.9 (55)	78.9 (15)	0.42
Ever quit before? (% yes)	72.8 (59)	94.7 (18)	0.07
Timing of COVID-19 Survey			
No. of days since COVID-19 impact	35.4 (15.1)		
No. of days since pre-COVID survey	83.9 (28.1)		
No. of months since quit attempt	7.3 (1.4)		
COVID-19 changes in living circumstances			
Changes in job situation^a^			
None	28.4 (23)		
Work from home (full or part time)	34.6 (28)		
Overtime	4.9 (4)		
Pay cut but not total loss	16.0 (13)		
Total loss	18.5 (15)		
Changes in living situation^a^			
None	75.3 (61)		
Consolidated (family/friend moving in/home)	14.8 (12)		
Roommate/family moved out	6.2 (5)		
Restrictions			
School closure	54.3 (44)		
Nonessential business closure	82.7 (67)		
Stay-at-home orders	74.1 (60)		
Personal contact with COVID-19^a^			
Infected with COVID-19 (incl. untested)	12.3 (10)		
Possibly exposed to COVID-19	14.8 (12)		
Family/friend/acquaintance hospitalized or died	14.8 (12)		

^a^ Does not sum to 100% due to unique changes (e.g., stopped volunteer work, health aide no longer comes, and house burnt down) and multiple responses (check all that apply).

SD, standard deviation.

### Self-rated impact of COVID-19

Most participants reported COVID-19-related changes in their living circumstances (Table 1), indicating partial or total loss of income, and transition to work from home. COVID-19-related restrictions affected most participants through nonessential business closures and stay-at-home orders, and several participants reported personal contact with the disease.

As shown in Table 2, participants rated their motivation to quit smoking or stay quit as a little less than “slightly” increased [*t*(80) = 3.65, *p* < 0.001], although more so in those who were abstinent post-COVID than those who were not [*t*(66) = −3.14, *p* = 0.0026]. Overall, only 17% of participants endorsed “same” for this item, 25% reported reduced motivation, and 58% reported increased motivation, with the modal response being “much more” (31%). The average increase in motivation to quit was significantly smaller than increases in worry [*t*(80) = −2.72, *p* < 0.01] and fear [*t*(80) = −2.01, *p* < 0.05], or items capturing civic engagement (e.g., news consumption [*t*(80) = −3.69, *p* < 0.001], contemplating future [*t*(80) = −3.17, *p* < 0.01], and desire to support the greater good [*t*(80) = −2.03, *p* < 0.05)]. Motivation to quit smoking increased significantly more than did eating healthy meals [*t*(80) = 2.73, *p* < 0.01]. Changes in motivation to quit smoking/stay quit were most strongly correlated with changes in prosocial or wellness promoting activities, but not significantly correlated with changes in fear, worry, stress, or depression.

**Table 2.** Descriptive Statistics on the COVID-19 Impact Measure

Since COVID-19 made a noticeable impact on my life, I have …	Mean (SD)	% Same	*p* ^a^	*r* ^b^
… watched, read or discussed the news.	1.7 (1.6)	12.3	0.00	0.26^*^
… spent time contemplating about what I want the future to look like.	1.6 (1.2)	19.8	0.00	0.05
… worried.	1.5 (1.5)	6.2	0.01	0.08
… felt afraid.	1.3 (1.5)	12.3	0.05	0.09
… felt a desire to support the greater good.	1.3 (1.4)	21.0	0.05	0.39^**^
… been bored.	1.1 (1.7)	14.8	0.32	−0.08
… been distracted from the tasks in my daily life.	1.1 (1.8)	11.1	0.39	−0.03
… gone out of my way to help others.	1.1 (1.4)	21.0	0.24	0.43^***^
… felt supported by the important people in my life.	1.0 (1.7)	24.7	0.46	0.27^*^
… felt lonely and isolated.	1.0 (1.5)	23.5	0.51	0.04
… felt gratitude.	1.0 (1.7)	22.2	0.51	0.21
… felt work-related stress.	1.0 (1.8)	23.5	0.62	0.04
… felt depressed.	0.9 (1.8)	28.4	0.90	−0.05
**… been motivated to quit smoking/stay quit.**	**0.8** (**2.0**)	**17.3**	**[Ref]**	**1.00**
if 30-day abstinent, … been motivated to quit smoking/stay quit.	1.4 (1.7)	22.2	—	—
if not 30-day abstinent: … been motivated to quit smoking/stay quit.	0.1 (2.1)	11.1	—	—
… deliberately engaged in activities to foster my mental well-being.	0.8 (1.4)	25.9	0.96	0.35^**^
… meaningfully engaged/interacted with my family.	0.8 (1.9)	14.8	0.81	0.34^**^
… found that it was hard for me to pay for the very basics like food, housing, medical care, and heating.	0.8 (1.5)	35.8	0.81	0.19
… have experienced conflict at home.	0.7 (1.7)	29.6	0.68	−0.03
… taken care of my physical well-being (exercise, sleep).	0.5 (1.8)	24.7	0.25	0.29^**^
… experienced help or kindness from strangers, neighbors, or acquaintances.	0.4 (1.4)	30.9	0.11	0.17
… eaten healthy meals.	0.1 (1.7)	24.7	0.01	0.29^**^
… felt optimistic that society can change in positive ways.	0.0 (1.9)	19.8	0.00	0.34^**^
… considered arming or took action to arm myself to protect myself and loved ones.	−0.1 (1.9)	34.6	0.00	0.23^*^
… meaningfully engaged/interacted with my friends.	−0.1 (2.1)	14.8	0.00	0.30^**^

Bold text indicates smoking cessation item.

Underlined text marks sub-group descriptives for this item, showing the means for those who were abstinent versus those who were not, respectively.

Likert scale ranges from −3 to 3, where −*3 = much less,* −*2 = moderately less,* −*1 = slightly less, 0 = same, 1 = slightly more, 2 = moderately more,* and *3 = much more*.

^a^ Significance of paired *t*-test comparing against “motivated to quit smoking/stay quit.”

^b^ Pearson's correlation between each item with “motivated to quit smoking/stay quit.”

^*^ *p* < 0.05, ^**^p < 0.01.

### Pre–post changes in smoking, emotional well-being, and other substance use

Per Table 3, self-reported 30-day abstinence rates and readiness to quit did not differ significantly pre versus post the onset of the COVID-19 outbreak. For the 45 who were not abstinent, there was no significant change in the number of cigarettes smoked in the past 30 days or the number of days smoked. Ever use of e-cigarettes increased, but current use of e-cigarettes was not elevated after the COVID-19 outbreak compared with before. There were significant pre–post increases in stress and negative affect and decreases in coping and positive affect. There was no significant change in reports of any drinking, but fewer participants reported exceeding NIAAA drinking guidelines and fewer participants reported heavy drinking.

**Table 3.** Within-Person Differences Pre- and Post-COVID-19 Onset

	Pre-COVID survey^a^ all (*n* = 100)	Pre-COVID survey^a^ postsample (*n* = 81)	COVID-19 survey (*n* = 81)	Pre–post effects for binary outcomes^b^	Pre–post effects for continuous outcomes^b^	Pre–post tests
	Mean/% (SD/*n*)	Mean/% (SD/*n*)	Mean/% (SD/*n*)	OR [95% CL]	*b* [95% CL]	*p*
Smoking						
30-Day self-reported abstinence (in %)	55.0 (55)	59.3 (48)	55.6 (45)	0.92 [0.63 to 1.33]		0.65
If not abstinent, recent cigarette use						
No. of days smoked (past 30 days)^c^	15.4 (10.9)	15.2 (11.0)	15.2 (12.3)		−0.11 [−4.21 to 3.99]	0.96
No. cigs per smoking day (past 30 days)^c^	4.6 (5.7)	4.8 (6.3)	4.6 (5.3)		−0.04 [−1.78 to 1.70]	0.96
Readiness to quit (% postcontemplation)^d^	86.0 (86)	90.1 (73)	81.5 (66)	0.65 [0.35 to 1.20]		0.16
Mental health						
PSS—stress	1.8 (1.0)	1.7 (1.0)	2.2 (1.0)		0.46 [0.28 to 0.64]	<0.0001
PSS—coping	2.5 (0.9)	2.6 (0.9)	2.3 (0.9)		−0.30 [−0.50 to −0.10]	0.0042
PANAS—positive affect	3.4 (0.9)	3.5 (0.8)	2.9 (1.0)		−0.59 [−0.80 to −0.37]	<0.0001
PANAS—negative affect	2.0 (1.0)	1.9 (1.0)	2.7 (1.1)		0.68 [0.47 to 0.88]	<0.0001
Other substance use						
Alcohol use (past 30 days)						
Any drinking (%)	34.0 (34)	34.6 (28)	29.1 (23)	0.82 [0.53 to 1.26]		0.35
Exceeded NIAAA drinking guidelines (%)	27.0 (27)	25.9 (21)	11.4 (9)	0.40 [0.22 to 0.71]		0.0018
Engaged in heavy drinking (%)	24.0 (24)	22.2 (18)	10.1 (8)	0.38 [0.19 to 0.76]		0.0061
E-cigarette use						
Ever (in %)	39.0 (39)	38.3 (31)	49.4 (39)	1.55 [1.02 to 2.36]		0.0417
Current (in %)	11.0 (11)	9.9 (8)	12.7 (10)	1.23 [0.70 to 2.14]		0.47

^a^ The most recent survey before the experience of COVID-19 impact in daily life (March 12, 2020 or participant named date, whichever was sooner) was used, which could be either the 6-month (51%) or 3-month (47%) or 6-week (2%) surveys.

^b^ GEE analysis and linear mixed effects models were used for longitudinal analyses, where TIME (0 = pre, 1 = post) was the independent variable of interest.

^c^ Among participants reporting nonabstinence at the pre-COVID survey only (*n* = 45).

^d^ Based on stages of change, contrasting preparation, action, and maintenance versus precontemplation and contemplation.

CL, confidence interval; GEE, generalized estimating equation; NIAAA, National Institute on Alcohol Abuse and Alcoholism; PANAS, Positive and Negative Affect Scale; PSS,Perceived Stress Scale.

## Discussion

The results of this survey provide initial insight into the overall impact of the COVID-19 pandemic on smokers in the process of undergoing a quit attempt in the first wave of the pandemic in the United States. As expected, emotional well-being was an important issue for study participants after the onset of the pandemic, and participants reported increases in worry, fear, and stress, similar to trends reported for the general population.^1–4^ Yet intriguingly, changes in worry, fear, stress, depression, and conflict were not correlated with changes in motivation to quit smoking.

Motivation to quit smoking remained relatively impervious to the outbreak of the COVID-19 pandemic, at least in the ∼1 month after participants experienced a noticeable impact on their daily lives. By that time, most participants had experienced changes in their jobs, loss of income, or household members moving, and 15% reported having a family member, friend or acquaintance hospitalized or die due to COVID-19. Despite these substantial impacts, smokers rated their motivation to quit smoking and stay quit, on average, as only slightly impacted, with the pre–post tests showing no significant change in abstinence rates, readiness to quit or, in the subset who still smoked, smoking frequency or amount. These findings are in line with previous reports of smoking behavior during the early phase of the pandemic, which indicated that, on average, the majority of current smokers or tobacco and electronic cigarette dual users at the onset of the pandemic did not change their smoking behavior during the pandemic.^9,14,34^ In extension to these previous findings, our results suggest that smokers undergoing quit attempts may also be less impacted by the COVID-19 pandemic than one might have expected. Of note, participants in this study were nondaily smokers, whose smoking tends to be more influenced by environmental and social cues than daily smokers'^35,36^ cues that were particularly impacted by COVID-19 social distancing measures. Moreover, a particularly important factor in nondaily smoking is mood^37^ that substantially shifted pre versus post the COVID-19 outbreak in this study, although not during the same follow-up timeframe in smokers using a prior version of the app before the pandemic.^36^ In a survey study in Australia during the early phase of the pandemic (April 9–19, 2020; *n* = 1491),^38^ increases in smoking were relatively uncommon (6.9%) but significantly associated with more severe depression, anxiety, and stress symptoms. In light of the observed mood shift in our study, it is surprising that the smoking behaviors of the nondaily smokers in this study were largely unaffected.

The overall trend for increased motivation to quit, however, masks substantial heterogeneity. Although on average the motivation to quit was only slightly increased, nearly a third of participants (31%) indicated that their motivation to quit was “much more” now compared with before the COVID-19 outbreak. The smoking cessation motivation of the vast majority of smokers was impacted, but these effects (58% reporting increased, 25% reporting decreased motivation) canceled each other out in averages. These findings are similar to those observed for current dual tobacco and electronic cigarette users (∼15% decreased, ∼48% the same, and ∼37% increased motivation to quit),^14^ and broadly reflect changes in smoking behavior among current smokers in the early phase of the pandemic.^14,34^ Overall, this heterogeneity in smoking responses to the onset of the pandemic reflects the complexity of competing motivators and stressors affecting current and recently quit smokers, where even net increases in stress have been linked to both increased and decreased motivation to quit smoking and smoking behavior.^34^

The smoking cessation approach used in our study explicitly promoted prosocial and wellness behaviors to bring about maintenance of positive affect in times of trouble. It is possible that engaging in positive psychology exercises for the period of 7 weeks right before the COVID-19 outbreak may have disproportionally prepared our participants for handling stressors without resorting to smoking. In support of this hypothesis, we noted very strong correlations between changes in motivation to quit and changes in prosocial and wellness behaviors. This finding suggests that it may be particularly fruitful to amplify these tendencies to enhance motivation to quit/stay quit during times of trouble.

We also examined alcohol and e-cigarette use. Despite news reports of increased alcohol sales,^39^ our data suggest that the nondaily smokers we surveyed decreased their engagement in problematic drinking. E-cigarette use appeared to be unaffected, too, although there was some indication of increased experimentation, with more participants indicating “ever” use, which did not translate to increases in “current use.” It is possible that the increase in the percentage of participants ever using e-cigarettes simply reflects the continued spread and prevalence of e-cigarette use independent of the pandemic, including as a tool to support quitting combustible cigarette use.

Several factors limit the generalizability of our findings. First, our sample size is relatively small and part of a prospective feasibility study without a control condition, with limited power to detect small effects and limited generalizability. Second, this survey was disproportionately completed by women and those who smoked more at baseline. Third, our COVID-19 impact scale was not psychometrically tested before survey administration. We developed this scale rapidly to assess the important early impact of the COVID-19 outbreak on smokers participating in smoking cessation trials and analyzed only individual items. Fourth, the study focused on nondaily smokers who differ from daily smokers on numerous characteristics related to smoking cessation.^40,41^ COVID-19 impact on trials involving daily smokers may be different.

## Conclusion

In conclusion, our results provide initial insight into the impact of the COVID-19 pandemic onset on nondaily smokers in a smoking cessation trial in the United States. Although emotional well-being was substantially impacted by the COVID-19 outbreak, self-reported smoking abstinence and readiness to quit were not negatively impacted. It may be that smoking cessation approaches targeting wellness and prosocial behavior may be particularly helpful to maintain and enhance motivation to quit smoking in the midst of a pandemic.

## Supplementary Material

Supplemental data

## Supplementary Material

Supplementary Appendix SA1

## Abbreviation Used

NIAAA: National Institute on Alcohol Abuse and Alcoholism
