# Supplementary material for: COVID-19 Impact on Smokers Participating in Smoking Cessation Trials: The Experience of Nondaily Smokers Participating in a Smartphone App Study
Source: Telemed Rep. 2021 Jun 14;2(1):179–87. doi: 10.1089/tmr.2021.0008 (PMC8812286; doi:10.1089/tmr.2021.0008)
Supplement: Supplemental data [file Supp_AppendixSA1.docx]

**COVID-19 Impact Scale**

1. **Pandemic-related psychological health**

Since when did COVID-19 (aka, the “corona virus”) make a noticeable impact on your life: ____________ [MM-DD-YYYY]

Since then, have you done/experienced the following things more, less, or about the same as compared to the time during the month before that date? Please use the following scale to describe the change:

○ ○ ○ ○ ○ ○ ○

-3 -2 -1 0 1 2 3

Much Moderately Slightly Same Slightly Moderately Much

less less less more more more

“Since COVID-19 made a noticeable impact on my life, I have …”

1. … felt supported by the important people in my life.
2. … found that it was hard for me to pay for the very basics like food, housing, medical care, and heating.
3. … meaningfully engaged/interacted with my family.
4. … meaningfully engaged/interacted with my friends.
5. … felt afraid.
6. … worried.
7. … felt gratitude.
8. … been distracted from the tasks in my daily life.
9. … watched, read or discussed the news.
10. … taken care of my physical well-being (exercise, sleep).
11. … felt optimistic that society can change in positive ways.
12. … considered arming or took action to arm myself to protect myself and loved ones.
13. … spent time contemplating about what I want the future to look like.
14. … felt lonely and isolated.
15. … eaten healthy meals.
16. … deliberately engaged in activities to foster my mental well-being.
17. … experienced help or kindness from strangers, neighbors, or acquaintances.
18. … felt work-related stress.
19. … have experienced conflict at home.
20. … felt depressed.
21. … gone out of my way to help others.
22. … been bored.
23. … felt a desire to support the greater good.
24. … been motivated to quit smoking / stay quit.

**B. Pandemic-related life events and change in circumstances**

To better define any significant changes you may have experienced, please complete the checklists below to indicate which of the possible impacts due to the COVID-19 pandemic affected you.

*Since the beginning of the COVID-19 crisis in the US (est. January 1, 2020), have you*:

1. Experienced any changes in your job situation (please check all that apply):

- None
- Worked from home, part-time (only check if this is a CHANGE for you)
- Worked from home, full-time (only check if this is a CHANGE for you)
- Worked overtime (>10 hours/week more than what's typical for me)
- Experienced a pay-cut, but not a total loss of income (i.e., if self-employed: reduction in business; if employed by others, took a cut in hours or pay)
- Lost your job or permanently closed your business (i.e., total loss of income)
- Started a new job
- Other (please describe: _________________________________________)

1. Experienced a change in your living situation (please check all that apply):

- None
- A dependent (e.g., child) moved back home
- Another friend or family member moved in with you
- You moved back in with parents or other family members
- A friend, roommate, or family member moved out
- Other (please describe: _________________________________________)

1. Have you been impacted by government restrictions (including town, state, and federal regulations and/or advisories)? Please check all that apply:

- None
- School closure
- Large gathering (> 50 people) restrictions
- Medium gathering (>10 people) restrictions
- Small gathering restrictions (>2 people restrictions, excluding direct family)
- Restaurants and bars no longer allow customers to dine in or stay
- Non-essential business closures (i.e., all businesses except for grocery stores, pharmacies, doctor’s offices, etc. are closed to the public)
- Lock-down order (i.e., shelter-in-place or stay-at-home orders)
- Medical quarantine due to travel
- Other (please describe: __________________________________________)

1. Your contact with COVID-19 includes (please check all that apply):

- You experienced symptoms of COVID-19 (e.g., fever, cough, sore throat, runny nose) but were not tested.
- You tested positive for COVID-19 but needed no hospitalization.
- You tested positive for COVID-19 and needed hospitalization.
- You were exposed or possibly exposed to COVID-19 but did not get infected.
- Someone you live(d) with tested positive for COVID-19.
- Someone you live(d) with was exposed or possibly exposed to COVID-19 but did not get infected.
- Someone in your circle of family, friends, and acquaintances tested positive for COVID-19 (but you did not live with them).
- Someone in your circle of family, friends, and acquaintances was hospitalized due to COVID-19.
- Someone in your circle of family, friends, and acquaintances died from COVID-19.

Lastly, please share any thoughts you have regarding COVID-19 and quitting smoking.

________________________________________________________________________________________________________________________________________________________________________________________________________________________________________________________________________________________________

**ADMINISTRATIVE USE ONLY**

Date form completed: / /

M M / D D / Y Y Y Y

Reviewer Initials:

Comments:
